# Multivariate analysis of food consumption profiles in crisis settings

**DOI:** 10.1371/journal.pone.0283627

**Published:** 2023-03-24

**Authors:** Aleksandra Gorzycka-Sikora, Nancy Mock, Michelle Lacey

**Affiliations:** 1 Department of Mathematics, University of Miami, Miami, Florida, United States of America; 2 Department of Global Community Health and Behavioral Sciences, Tulane University, New Orleans, Louisiana, United States of America; 3 Department of Mathematics, Tulane University, New Orleans, Louisiana, United States of America; Tabriz University of Medical Sciences, IRAN, ISLAMIC REPUBLIC OF

## Abstract

Preventing malnutrition is one of the primary objectives of many humanitarian agencies, and household surveys are regularly employed to monitor food insecurity caused by political, economic, or environmental crises. Consumption frequencies for standard food groups are often collected to characterize the depth of food insecurity in a community and measure the impact of food assistance programs, producing a vector of bounded, correlated counts for each household. While aggregate indicators are typically used to summarize these results with a single statistic, they can be difficult to interpret and provide insufficient detail to judge the effectiveness of assistance programs. To address these limitations, we have developed a multivariate modeling framework for consumption frequency data. We introduce methods to update baseline models for the analysis of the smaller and more variable surveys typically collected in crisis settings, and we present an application of our approach to national consumption data collected in Yemen in 2014 and 2016 by the World Food Programme. The approach provides more nuanced and interpretable information about consumption changes in response to shocks and the effectiveness of humanitarian assistance.

## Introduction

One of the 17 Sustainable Development Goals identified by the United Nations in 2015 is to achieve Zero Hunger by 2030, ensuring “access by all people, in particular the poor and people in vulnerable situations, including infants, to safe, nutritious and sufficient food all year round” [1]. However, society is currently far from meeting this ambitious objective, with nearly 30% of all people estimated to suffer from moderate to severe food security worldwide in 2020 [2]. Food insecurity has many causes, some associated with chronic poverty and others related to shocks such as conflict, economic collapse, disease outbreak, or environmental disasters. The role of the UN World Food Programme (WFP) is to address food insecurity by providing emergency assistance as well as working to improve resilience in the long-term. The collection and analysis of data to support monitoring and evaluation efforts are critical components of the agency’s mission.

WFP and its partners rely heavily on direct input from the residents of affected communities, typically acquired through surveys conducted either in-person, by phone, or, more recently, through text messaging. A common tool is the Comprehensive Food Security and Vulnerability Assessment (CFSVA), a lengthy survey that is conducted periodically in nations where WFP has active programs. While questions vary based on context, one of the modules routinely included in a CFSVA is designed to obtain standard information about weekly food consumption. Respondents are asked to state the number of days in the previous week on which the members of their household consumed foods from a standard list of groups, based on the assumption that meals in the household are communal. The Food Consumption Score (FCS), initially developed by WFP in 1996, is a summary statistic intended to measure the adequacy of the diet from both a nutritional and caloric standpoint [3]. It is computed by taking a weighted average of the days of consumption of 8 major food groups, defined as Starches, Vegetables, Fruits, Pulses (Beans, Peas, and Lentils), Dairy, Animal Proteins (Meat, Poultry, Seafood, and Eggs), Fats, and Sugar, according to the following formula: *FCS* = (*Starches* × 2) + (*Pulses* × 3) + *Vegetables* + *Fruit* + (*Meat* × 4) + (*Dairy* × 4) + (*Fats* × .5) + (*Sugar* × .5).

The weights sum to 16, and the maximum attainable score is 112. In its reporting, WFP often considers two thresholds for the FCS. Households scoring at or below 28 are considered to not be meeting minimal consumption levels, as a score this low would be associated with consuming little other than starches, fats, and sugar on a daily basis, while households scoring between 29 and 42 are said to have inadequate levels of consumption (see [4] for additional details).

An alternative food security metric, developed by the Food and Nutrition Technical Assistance Project (FANTA) is the Household Dietary Diversity Score (HDDS), which is defined as the sum of indicators for whether each of the following 12 food groups was consumed the previous day: Cereals, Roots and tubers, Vegetables, Fruits, Meat/poultry/offal, Eggs, Fish and seafood, Pulses/legumes/nuts, Milk and milk products, Oil/fats, Sugar/honey, and Miscellaneous (see [5] for additional details). The FCS and the HDDS have been studied extensively, both with respect to their methodological underpinnings and their correlation with other nutrition outcomes [6–12].

While the prevalence of food insecurity based on these indicators is widely reported in practice, the underlying data collected to produce the FCS and HDDS are a rich resource for analyzing dietary behavior that is generally not considered in depth. Agency reports may include summary statistics for individual food groups, typically limited to sample averages, but this provides little insight about the variation across households, the role of regional dietary preferences, or the correlations between various food groups. For these reasons, we were motivated to develop an approach for the joint analysis of the module count data, where the observation for each household is a vector of dependent integer-valued variables on the interval [0, 7]. Literature on methods for the analysis of multivariate count data is quite sparse, although an emerging application currently generating a great deal of research interest is in the context of the gut microbiome, where observed counts for bacterial taxa are highly dispersed (see, for example, [13]). More broadly, Bonat and Jørgensen have developed a general modeling framework for regression models with multivariate response variables from any exponential family distribution [14], implemented in the R package “mcglm” [15]. An example of the joint modeling of discrete consumption data is a study conducted by Rodrigues-Motta *et al.*, where the authors analyzed the distribution of insect types consumed by a Brazilian marsupial species based on fecal samples [16]. Their dataset was small (37 animals) and counts were generally very low with many zeroes, leading them to propose a zero-inflated Poisson model with Gaussian random effects terms that was estimated via maximum likelihood with numerical integration. For longitudinal binary data, Molenberghs *et al.* introduced a beta model with normal random effects to account for overdispersion as well as correlation across the observations for each subject [17], applying this approach to the analysis of school attendance and infant overweight status in Southwest Ethiopia using both maximum likelihood and Bayesian methods [18]. Our multivariate Bayesian beta-binomial model extends this framework to the bounded count data collected for food consumption survey datasets, jointly estimating parameters for daily consumption probabilities and dispersion as well as covariance terms to account for variability across households and the correlations among food groups.

In areas experiencing emergencies due to conflict, environmental disasters, or other factors causing severe stress, it is common for WFP and other aid agencies to conduct monitoring surveys to assess the extent to which the food security situation has deteriorated and to determine whether assistance programs are having an impact. Because these typically involve smaller numbers of households and are conducted under difficult circumstances, the data collected in such Emergency Food Security Assessments (EFSAs) are necessarily more variable than those obtained through routine surveys. However, this variability can be reduced by incorporating information from past models. We therefore have implemented a Bayesian updating approach, in which posterior distributions estimated from a previous comprehensive survey are incorporated as informative priors for a subsequent round. We apply our approach to an analysis of food consumption surveys in Yemen, first estimating baseline models for a CFSVA collected in 2014 [19] and subsequently analyzing changes in dietary patterns based on EFSA data collected two years later [20] following the intensification of conflict and a significant increase in economic turmoil. De-identified data and model code are available for download at https://github.com/mlacey1/Yemen_BBMods.git.

## Materials and methods

### Model specification

The beta-binomial distribution is defined for a discrete variable *Y* which takes integer values on the interval {0, *m*} for some fixed *m*. Conditional on a parameter *p*, *Y* follows a *Binomial*(*m*, *p*) distribution, while *p* follows a *Beta*(*α*, *β*) distribution for positive reals *α* and *β*. It follows directly that the marginal density of *Y* has the form

f(y;m,α,β)=(my)B(y+α,m-y+β)B(α,β)

where *B*(⋅, ⋅) is the beta function. The mean and variance of this beta-binomial random variable are given by

$$\begin{array}{c} E\left(Y\right)=\frac{m\alpha }{\alpha +\beta }\;\text{and}\;Var\left(Y\right)=\frac{m\alpha \beta (\alpha +\beta +m)}{{\left(\alpha +\beta \right)}^{2}\left(\alpha +\beta +1\right)}, \end{array}$$

respectively. An alternative parametrization of this model, which will be adopted here, defines *pθ* = *α* and (1 − *p*)*θ* = *β*, where *θ* > 0 and *p* ∈ (0, 1). This results in the more intuitive expressions

$$\begin{array}{c} E\left(Y\right)=mp\;\text{and}\;Var\left(Y\right)=mp\left(1-p\right)(1+\frac{m-1}{\theta +1}). \end{array}$$

In this context the parameter *θ* is interpreted as an overdispersion parameter, so that for large values of *θ* the variance converges to *mp*(1 − *p*), the variance of the Binomial distribution with parameters *m* and *p*.

In the multivariate case, we consider a vector of length *J* that contains counts {*Y*_1_, …, *Y*_*J*_}, each on the interval {0, *m*}. Each element *Y*_*j*_ is a random variable following a marginal beta-binomial distribution with parameters *p*_*j*_ and *θ*_*j*_. To allow correlation between elements *Y*_*j*_ and *Y*_*k*_, we incorporate normally distributed random effects. Given an *n* × *k* matrix of observations *Y*_*ij*_, *i* = {1, …, *n*}, *j* = {1, …, *J*}, *u*_*i*_ = {*u*_*i*1_, *u*_*i*2_, …, *u*_*iJ*_} are independent and identically distributed multivariate normal random variables with mean 0 and *J* × *J* covariance matrix *Σ*. Conditional on these random effects, we assume that the observations are independent:

$$\begin{array}{c} Y_{ij}|u_{ij}∼\text{Beta-Bin}\left(m,p_{j},\theta_{j}\right)\;\text{where}\;u_{i}∼N\left(0,\Sigma \right). \end{array}$$

We introduce this multivariate beta-binomial model to the recorded weekly consumption frequencies, where *Y*_*ij*_ is a random variable that denotes the number of days within the 7-day recall period on which household *i* consumed food group *j*. The parameter *p*_*j*_ denotes the probability of consumption each day, and *θ*_*j*_ is a dispersion parameter that controls how far the counts deviate from a standard binomial distribution. To account for household-to-household variability and correlation among the food groups, we adjust *p*_*j*_ using normally distributed random effects *κ*_*ij*_ where *σ*_*j*_ is the standard deviation associated with food group *j* and *ρ*_*jk*_ is the correlation in consumption between food groups *j* and *k*.

For the 7-day consumption surveys considered in the food security setting, our implementation of this model is as follows:

$$\begin{array}{c} Y_{ij}∼\text{Beta-Bin}\left(7,\gamma_{ij},\theta_{j}\right) \end{array}$$

where

$$\begin{array}{c} \gamma_{ij}=\frac{exp(log\left(\frac{p_{j}}{1-p_{j}}\right)+\kappa_{ij})}{1+exp(log\left(\frac{p_{j}}{1-p_{j}}\right)+\kappa_{ij})} \end{array}$$

and

$$\begin{array}{c} \kappa_{ij}∼N\left(0,\Sigma \right)\;\text{for}\;\Sigma =D\Omega D\;\text{with}\;D=Diag\left(\sigma_{1},\ldots ,\sigma_{J}\right), \end{array}$$

the vector of standard deviations for the random effects for each food group. Ω is the *J* × *J* correlation matrix. Household-level parameters *γ*_*ij*_ are defined such that *γ*_*ij*_ = *p*_*j*_ when *κ*_*ij*_ = 0 and 0 ≤ *γ*_*ij*_ ≤ 1 for all $\kappa_{ij}\in R$.

### Survey data

In 2014, the CFSVA was conducted in 19 regions, each with 480 households surveyed. Households were selected using a two-stage stratified sampling design, in which 16 households were randomly selected from each of 30 randomly selected enumeration areas within each region. Respondents were asked about their household consumption of 13 food groups: bread and wheat (henceforth denoted “bread”), potatoes and tubers (“potatoes”), rice and other starches (“rice”), vegetables, fruit, pulses, eggs, dairy, meat and poultry (“meat”), fish and seafood (“fish”), oils and fats (“oil”), sugar and sweets (“sugar”), and condiments. In 2016, the EFSA included a different sample of 360 households in each of the 19 regions (with the exception of the southwestern governorate of Taiz, where only 231 households participated).

### Parameter estimation

We fit multivariate beta-binomial models to our baseline 2014 surveys using a Bayesian framework, with the following diffuse priors:

$$\begin{array}{c} p_{j}∼\text{Beta}\left(1,1\right),\theta_{j}∼\text{Gamma}\left(5,1\right),\sigma_{j}∼\text{Half-Cauchy}\left(0,2\right),\;\text{and}\;\Omega ∼LKJ\left(2\right), \end{array}$$

the latter being a density on correlation matrices developed by Lewandowski *et al.* that makes use of the Cholesky factorization to substantially improve computational efficiency [21]. We note that the Beta(1,1) density is equivalent to a uniform distribution on the interval (0,1), while the Gamma(5,1) density is right-skewed with mean 5 and 99% of its mass concentrated on the interval (0, 11.6). Models were coded in the Stan language [22] and run in R with the RStan package [23]. Runs were conducted in parallel on a high-performance server using four chains for 4000 steps each, with the first 1000 discarded as burn-in.

### Bayesian updating

For the 2016 surveys, a Bayesian updating approach was employed, by which parameters estimated from the 2014 surveys formed a basis for priors for the subsequent round. Consider a baseline survey conducted on a sample of *n*_0_ households with response count vectors *X*_*i*_ = {*X*_*i*1_, …, *X*_*iJ*_}. The posterior distribution for the parameter *p*_0*j*_ will be

$$\begin{array}{c} \text{Beta}(1+\sum_{i=1}^{n_{0}}X_{ij},1+7n_{0}-\sum_{i=1}^{n_{0}}X_{ij}), \end{array}$$

with mean and variance given by

$$\begin{array}{c} \mu_{0j}=\frac{1+7n_{0}{\hat{p}}_{0j}}{2+7n_{0}}\;\text{and}\;\sigma_{0j}^{2}=(\frac{1+7n_{0}{\hat{p}}_{0j}}{2+7n_{0}})(\frac{1+7n_{0}\left(1-{\hat{p}}_{0j}\right)}{2+7n_{0}})(\frac{1}{3+7n_{0}}) \end{array}$$

respectively, where ${\hat{p}}_{0j}=\frac{\sum_{i}X_{ij}}{7n_{0}}$ is the sample proportion from the baseline survey for food group *j*. For large sample sizes, this distribution is highly constrained, and even for a sample of size 100 the posterior standard deviation is bounded above by 0.02. While narrow posterior distributions are generally desirable, the consequence of directly employing such a distribution as a prior for a subsequent round is that it highly biases the estimated posterior so that the new data are overly discounted. To correct this, we introduce a weighting parameter *a* so that the updated prior for a proportion *p*_*j*_ has the following form:

$$\begin{array}{c} p_{j}∼\text{Beta}(a(\frac{1+7n_{0}{\hat{p}}_{0j}}{2+7n_{0}}),a(\frac{1+7n_{0}-7n_{0}{\hat{p}}_{0j}}{2+7n_{0}}))\approx \text{Beta}(a{\hat{p}}_{0j},a(1-{\hat{p}}_{0j})). \end{array}$$

Applying this prior with a new dataset of *n*_*d*_ observations *Y*_*k*_ = {*Y*_*k*1_, …, *Y*_*kJ*_}, *k* = 1, …, *n*_*d*_, the updated posterior beta distribution for the parameter *p*_*dj*_ has approximate mean and variance

$$\begin{array}{c} \mu_{dj}\approx \frac{a{\hat{p}}_{0j}+7n_{d}{\hat{p}}_{dj}}{a+7n_{d}}, \end{array}$$

$$\begin{array}{c} \sigma_{dj}^{2}\approx (\frac{a{\hat{p}}_{0j}+7n_{d}{\hat{p}}_{dj}}{a+7n_{d}})(\frac{a\left(1-{\hat{p}}_{0j}\right)+7n_{d}-7n_{d}{\hat{p}}_{dj}}{a+7n_{d}})(\frac{1}{a+7n_{d}+1}). \end{array}$$

Larger values of *a* place greater weight on the prior estimates ${\hat{p}}_{0j}$ and result in posterior distributions with smaller variance, while smaller values produce more variable estimates that are less biased towards the prior. Similarly, for estimating updated dispersion parameters *θ*_*dj*_ we define a prior distribution of the form $\text{Gamma}(\alpha {\hat{\theta }}_{0j},\alpha ),$ which has mean ${\hat{\theta }}_{0j}$ and variance $\frac{{\hat{\theta }}_{0j}}{\alpha },$ so that larger values of *α* result in a highly constrained prior. While the joint posterior distribution of (*p*_*dj*_, *θ*_*dj*_) does not have a closed form, it has been established that under suitable regularity conditions the posterior distribution will be asymptotically normal with mean equal to the posterior mode and variance equal to the reciprocal of the second derivative of the logarithm of the likelihood function evaluated at the posterior mode [24]. These moments can be computed numerically, and we have shown that the normal approximation is reasonably accurate for sample sizes *n*_*d*_ ≥ 250 (see [25] for details). With this result, it is possible to estimate boundaries for the high posterior density (HPD) regions associated with choices of weights *a* and *α* for a given sample size *n*_*d*_, as well as determining the bias in estimation of the posterior mode for specific pairs (*p*_0*j*_, *θ*_0*j*_) and (*p*_*dj*_, *θ*_*dj*_).

For the Yemen surveys, weights *a* = 50 and *α* = 5 were chosen based on our normal approximations, which indicated that 95% HPD regions would be centered at the posterior mode with width bounded by ±0.025 for *p*_*dj*_ and ±2.2 for *θ*_*dj*_ values less than 10 (beyond which the beta-binomial is practically indistinguishable from the regular binomial distribution in this setting). Based on previous results [25], priors for correlation terms were set to be normally distributed with means set to 2014 estimates and variance 1, truncated to the (-1,1) interval, while priors for the standard deviations remained *Half*−*Cauchy*(0, 2).

### Assessing goodness of fit

The goodness of fit of the model to the survey data in each region was assessed by comparing the distributions of simulated data from each fitted model in two ways: (1) Univariate analysis for each food group, based on nonparametric tests for center and variability (Wilcoxon Rank-sum and Fligner-Killeen, respectively), and (2) Similarity of the distributions of the FCS and a frequency-weighted HDDS, evaluated using Kolmogorov-Smirnoff tests. Because our surveys represent weekly consumption frequencies, the weighted HDDS was computed by dividing the counts for each food group by 7 to obtain a daily average for each component of the indicator. Test *p*-values were adjusted for multiple comparisons using the Benjamini-Hochberg procedure.

## Results

**Fig 1:**
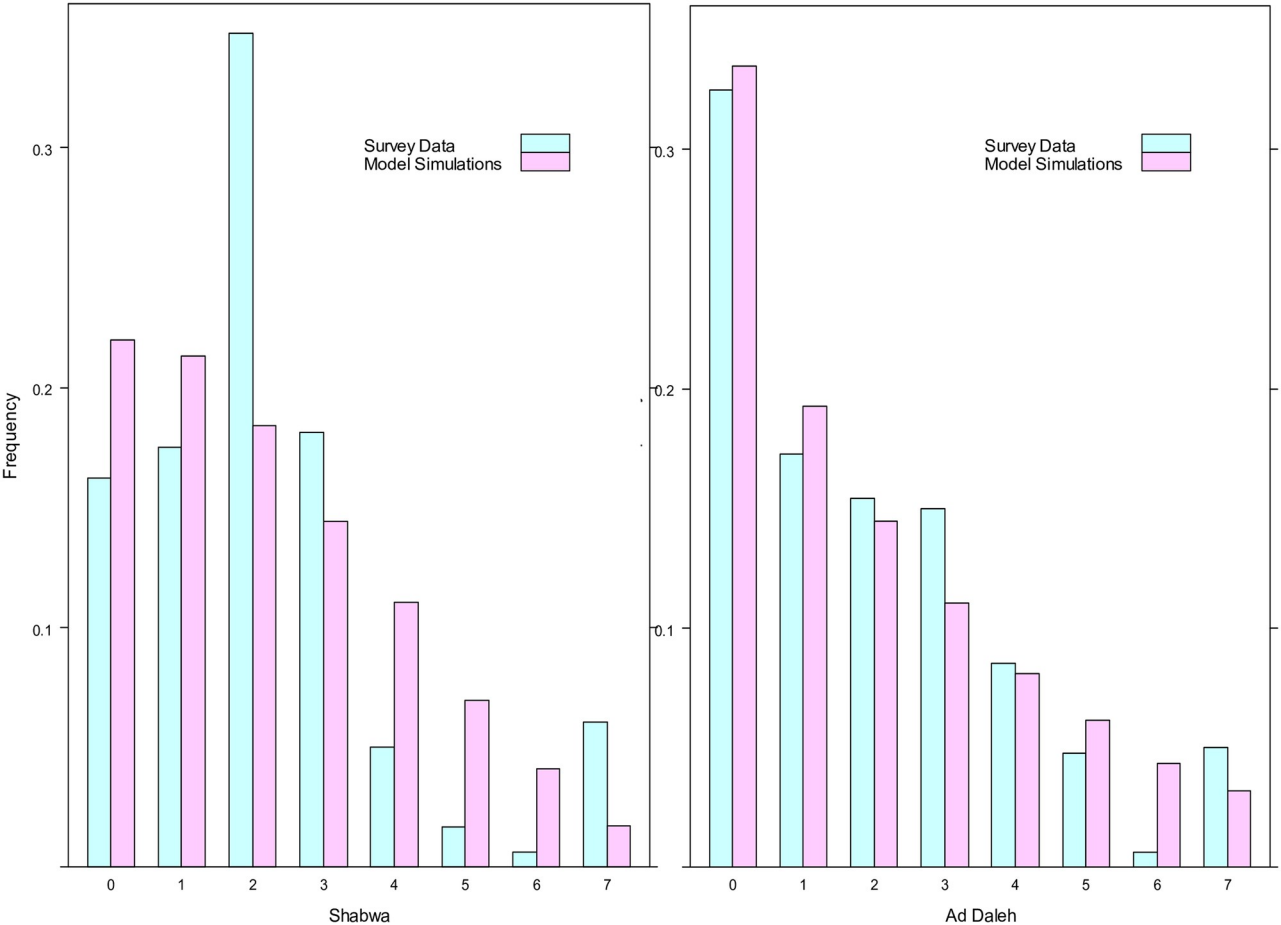
Empirical distributions and fitted beta-binomial models for vegetable consumption.

For the 2014 data, there were no statistically significant differences in median between the 480 observations in each region surveyed and the 12,000 data points generated by the model simulation for any of the 13 food groups considered. And, for the majority of food groups, there was very good concordance in variability between the survey data and simulations, with statistically significant differences observed in either one or none of the regions for 10 of the 13 food groups (bread, rice, fruit, pulses, eggs, dairy, fish, oil, sugar, and condiments). However, there were widespread differences for potatoes (10 regions), vegetables (6 regions), and meat (13 regions). Upon inspection, we determined that these discrepancies were due to atypical or erratic distributions in the survey data for these food groups. For example, in both Shabwa and Ad Daleh the average consumption for vegetables was 2 days per week. While this corresponded to a right-skewed distribution in Ad Daleh that was well-approximated by the beta-binomial model, in Shabwa there was an unusual response profile with a very high frequency (35%) at exactly 2 days that could not be replicated by the fitted model (see Fig 1). For meat, many of the regional models were unable to reproduce high frequencies at the value “1,” which likely reflects the Islamic tradition of eating meat once per week, while for potatoes/tubers many of the response profiles were multi-modal with three or more peaks.

**Fig 2:**
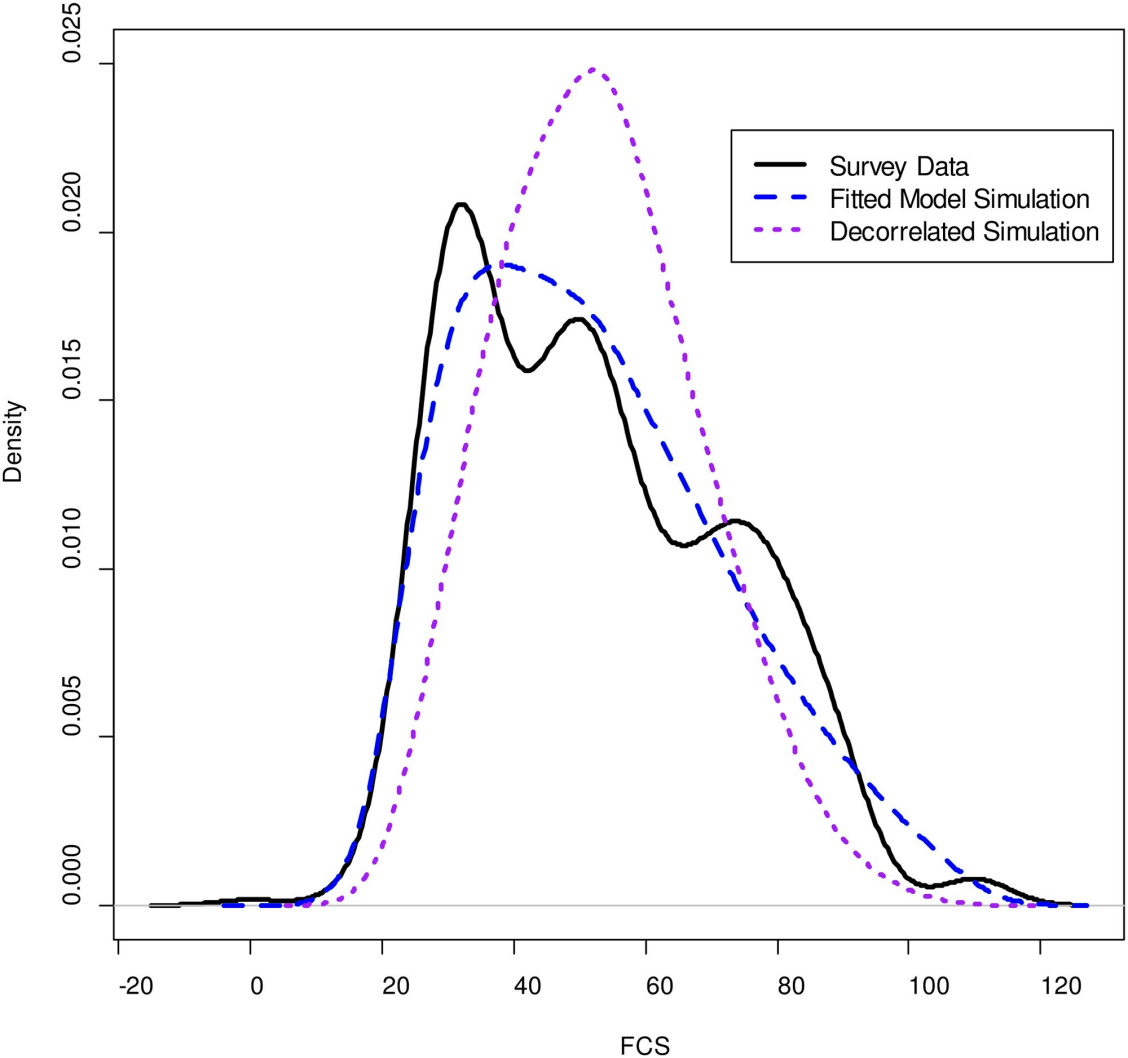
Comparison of beta-binomial and decorrelated model fits to survey data in the Al Jawf governorate.

After assessing the goodness of fit for the individual food groups, we considered the ability of the simulated data to reproduce the FCS and weighted HDDS statistics. This is essential for our analyses, not only because these statistics are important for food security reporting but because they reflect on the ability of the model to capture household variation through the inclusion of correlated random effects. To illustrate, Fig 2 displays kernel density estimates for the FCS distribution of the 480 surveyed households along with the FCS calculated from a simulated dataset derived from the fitted model. While the simulated distribution (which is based on 12,000 data points) is clearly much smoother than the original data, it captures the overall right-skewed shape of the distribution due to a high correlation in this region in the consumption of “high-value” foods such as meat, eggs, and dairy. If we were to ignore these correlations and simply compute the FCS based on independent samples from each food group we would obtain the far more symmetric distribution also shown in the figure, which has the same mean as the others but clearly does not capture the high level of consumption inequality evident in the data.

Overall, among the 19 regions only one (Mareb) showed a statistically significant deviation from the empirical household distribution for the FCS according to the K-S test (adjusted *p*-value = 0.046), and for another region (Hudeida) the model simulation failed to accurately reproduce the weighted HDDS distribution (adjusted *p*-value = 0.008). There were no regions with questionable results for both the FCS and the HDDS.

Using our novel Bayesian updating approach, models for 2016 were fit using prior distributions for *p*_*j*_, *θ*_*j*_ and *ρ*_*jk*_ centered at the parameter values estimated for the 2014 surveys.

Goodness of fit results for the 19 regions showed no significant deviations in median between the observed and simulated distributions for any food group (Wilcoxon rank-sum tests), with some discrepancies in variation detected for potatoes/tubers (3 regions), vegetables (6 regions) and meat (5 regions) as seen in 2014 (Fligner-Killeen tests). There were also 5 regions where reported rice consumption was significantly more erratic than could be well-approximated by the beta-binomial model, a phenomenon that was not observed in 2016. At the indicator level, none of the simulated FCS distributions were inconsistent with the 2016 survey data (Kolmogorov-Smirnoff tests with *p*-values adjusted using the Benjamini-Hochberg procedure), although the simulated weighted HDDS distributions were significantly different from the 2016 data in 5 regions due to the presence of a few extreme values among the surveyed households.

As a diverse nation with a population of 26 million, regional context is important to consider when analyzing food security and dietary trends. In 2014, WFP estimated that nationally 41% of Yemenis were food insecure overall with 19% severely insecure, but this varied considerably at the regional level [19]. We illustrate these differences through an overview of our results in 6 distinct regions with varying food security scenarios both before and after the crisis.

### Ibb

**Fig 3:**
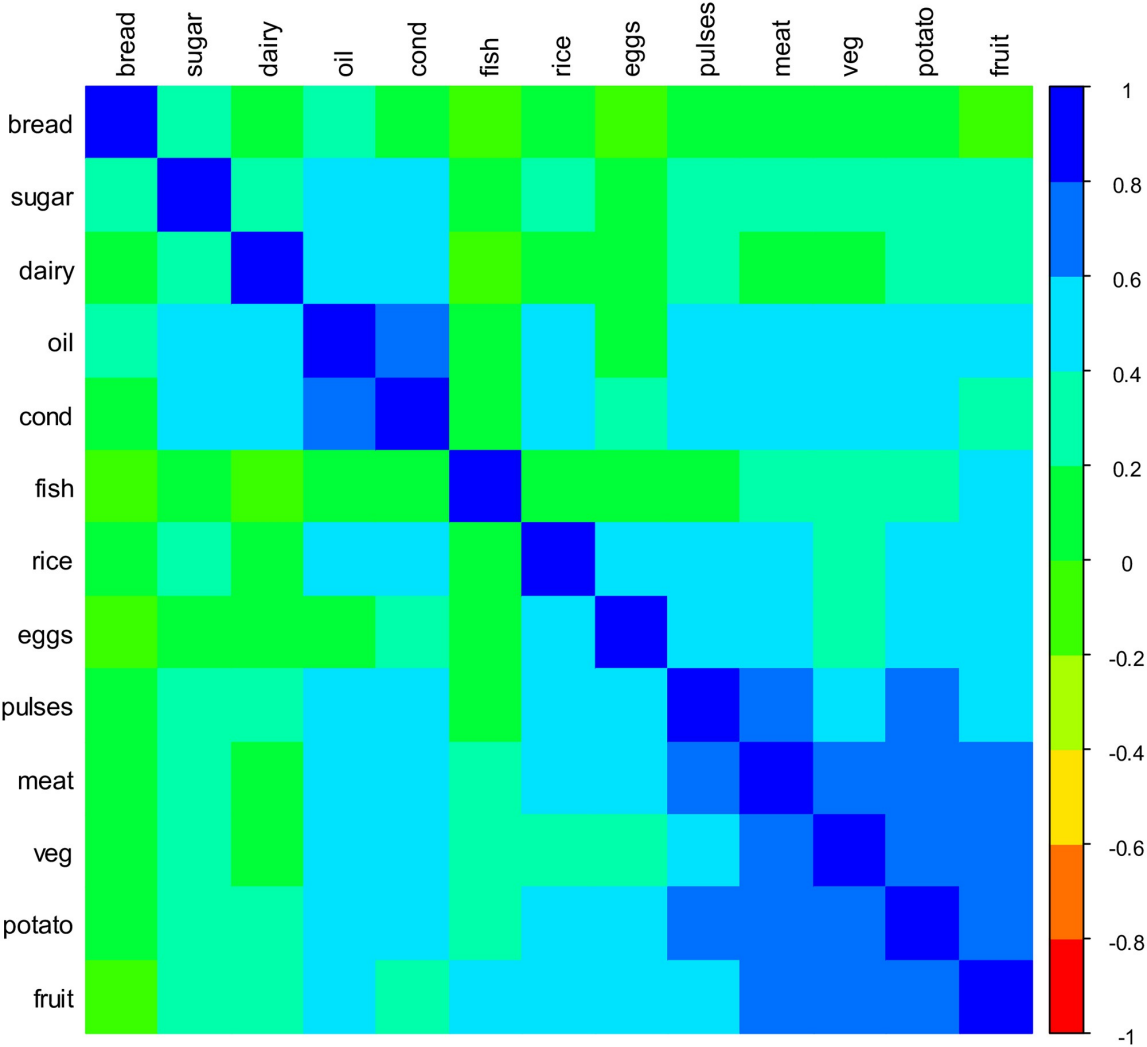
Estimated random effects correlation matrix for Ibb in 2014.

We begin with Ibb, a populous area in the dry highlands. In 2014, 19.5% of surveyed households in Ibb were at or below the threshold for minimal consumption and 42.9% were below the threshold for adequate consumption, consistent with the national average. As in most of the country, bread, oil, sugar and condiments were consumed daily in nearly all households, but in this region there was only sporadic consumption of fruit, beans, eggs, and fish. Meat was consumed on at most one day for the large majority of households. Thus, while the median unweighted weekly HDDS value was 8 food groups (indicating any consumption during the entire week), after accounting for frequency weighting this dropped to 5.4. Vegetables and dairy were consumed more regularly (estimated daily consumption probabilities ${\hat{p}}_{j}$ were 0.40 and 0.23, respectively), but the fitted distributions for both of these food groups were both highly dispersed with respective ${\hat{\theta }}_{j}$ estimates 5.2 and 4.3. Most importantly, the fitted model incorporated a great deal of variation across households with strong correlations in consumption for many food groups. As illustrated by Fig 3, which graphically displays the magnitude of estimated correlation parameters for the random effects terms, households consuming less common foods such as fruit were much more likely to consume “high value” foods such as meat and pulses, while struggling households reduced consumption of both oil and condiments. Interestingly, dairy was largely uncorrelated with other food groups, likely due to the fact that nearly half of the households that consumed dairy stated that they had either produced it themselves (through livestock ownership) or received it as a gift.

**Fig 4:**
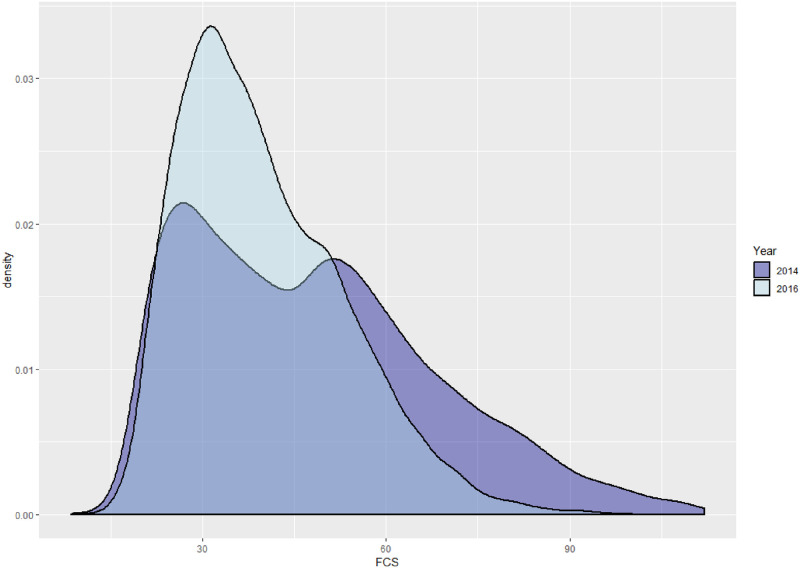
Density curves for simulated FCS distributions from fitted models in Ibb.

The strong correlations in household consumption patterns not surprisingly resulted in a highly skewed and bimodal distribution for the FCS in Ibb in 2014 (see Fig 4), likely reflecting a high degree of income inequality within the region. Thus while the “average” FCS score in the simulated distribution from the fitted model was 48, the lower quartile of households were below 32 and the upper quartile were above 60.

By 2016, conditions had significantly deteriorated in this region due to the ongoing conflict. While there was no significant increase in the proportion of households below minimal consumption levels (the 2016 sample proportion was 23.9%), the number of households reporting consumption below acceptable levels rose to 68%, a massive increase of over 25%. Analysis of the 2016 modeling results indicates that there were significant decreases in consumption of meat, vegetables, and potatoes, offset by an increase in consumption of pulses. While none of the distributions became significantly less dispersed, the variability across households declined for vegetables, pulses, eggs, dairy, and meat (see Table 1). These changes in consumption are reflected in a unimodal and far less skewed FCS distribution in 2016 (Fig 4), with a much narrower interquartile range of (31,48.5). The median unweighted weekly HDDS remained at 8 in 2016 although the median weighted HDDS decreased to 4.9, indicating an overall decline in consumption frequencies across the food groups.

**Table 1:** Parameter estimates for 2014 and 2016 models in Ibb.

Food Group	Year	Proportion *p*		Dispersion *θ*		Standard Deviation *σ*	
		Mean	95% HPD Int	Mean	95% HPD Int	Mean	95% HPD Int
Bread	2014	1.00	(0.99,1.00)	0.98	(0.32,2.20)	0.91	(0.05,1.98)
	2016	1.00	(0.99,1.00)	0.71	(0.27,1.44)	1.08	(0.07,2.14)
Potatoes	2014	0.23	(0.19,0.27)	8.50	(4.89,13.62)	1.77	(1.47,2.07)
	2016	0.14	(0.11,0.17) ↓	8.73	(6.48,11.37)	1.57	(1.29,1.88)
Rice	2014	0.62	(0.57,0.67)	5.58	(2.97,10.01)	1.77	(1.38,2.16)
	2016	0.54	(0.49,0.59)	5.34	(3.72,7.34)	1.66	(1.35,1.99)
Vegetables	2014	0.40	(0.34,0.45)	5.19	(2.74,8.96)	1.98	(1.54,2.39)
	2016	0.26	(0.23,0.29) ↓	7.39	(5.64,9.5)	0.96	(0.77,1.16) ↓
Fruit	2014	0.02	(0.01,0.03)	8.41	(4.45,13.87)	2.13	(1.67,2.63)
	2016	0.04	(0.03,0.05)	9.80	(7.47,12.48)	1.56	(1.24,1.91)
Pulses	2014	0.11	(0.08,0.15)	6.87	(3.50,11.66)	2.61	(2.12,3.10)
	2016	0.18	(0.16,0.2) ↑	7.82	(5.95,10.1)	0.53	(0.28,0.80) ↓
Eggs	2014	0.02	(0.01,0.04)	6.74	(3.25,11.76)	2.60	(1.98,3.2)
	2016	0.04	(0.03,0.05)	7.59	(5.53,9.98)	1.44	(1.06,1.85) ↓
Dairy	2014	0.23	(0.13,0.32)	4.29	(1.92,8.18)	3.70	(2.79,4.56)
	2016	0.15	(0.11,0.19)	4.08	(2.57,5.95)	2.10	(1.61,2.58) ↓
Meat/Poultry	2014	0.14	(0.13,0.16)	18.56	(12.99,25.96)	0.73	(0.58,0.88)
	2016	0.09	(0.08,0.11) ↓	20.80	(17.2,24.78)	0.32	(0.10,0.53) ↓
Fish/Seafood	2014	0.01	(0.00,0.02)	4.99	(2.14,10.07)	1.64	(0.64,2.62)
	2016	0.00	(0.00,0.01)	5.56	(3.77,7.71)	1.54	(0.73,2.31)
Oils/Fats	2014	1.00	(0.99,1.00)	1.01	(0.49,1.88)	3.07	(2.31,3.69)
	2016	0.98	(0.97,1.00)	1.54	(0.93,2.43)	2.88	(1.96,3.99)
Sugar	2014	1.00	(0.99,1.00)	0.92	(0.30,2.09)	1.66	(0.78,2.29)
	2016	1.00	(1.00,1.00)	0.82	(0.38,1.5)	2.53	(1.8,3.15)
Condiments	2014	1.00	(0.99,1.00)	0.69	(0.34,1.26)	4.12	(2.88,4.93)
	2016	1.00	(0.99,1.00)	1.28	(0.71,2.09)	3.53	(2.43,4.57)

Arrows indicate non-overlapping 95% HPD intervals across years with estimates increasing (↑) or decreasing (↓).

### Al Jawf and Al Mahweet

**Fig 5:**
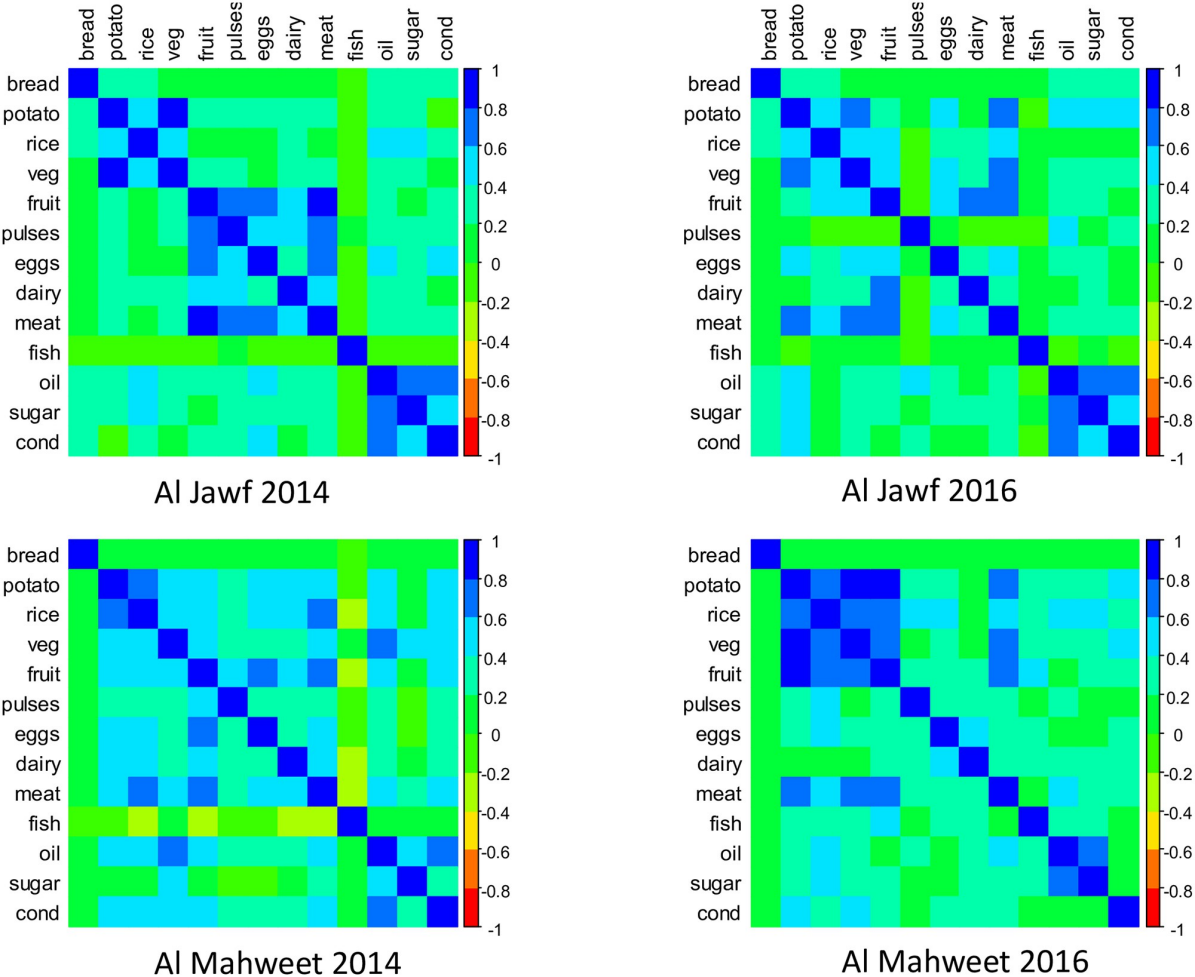
Estimated random effects correlation matrices in Al Jawf and Al Mahweet.

**Fig 6:**
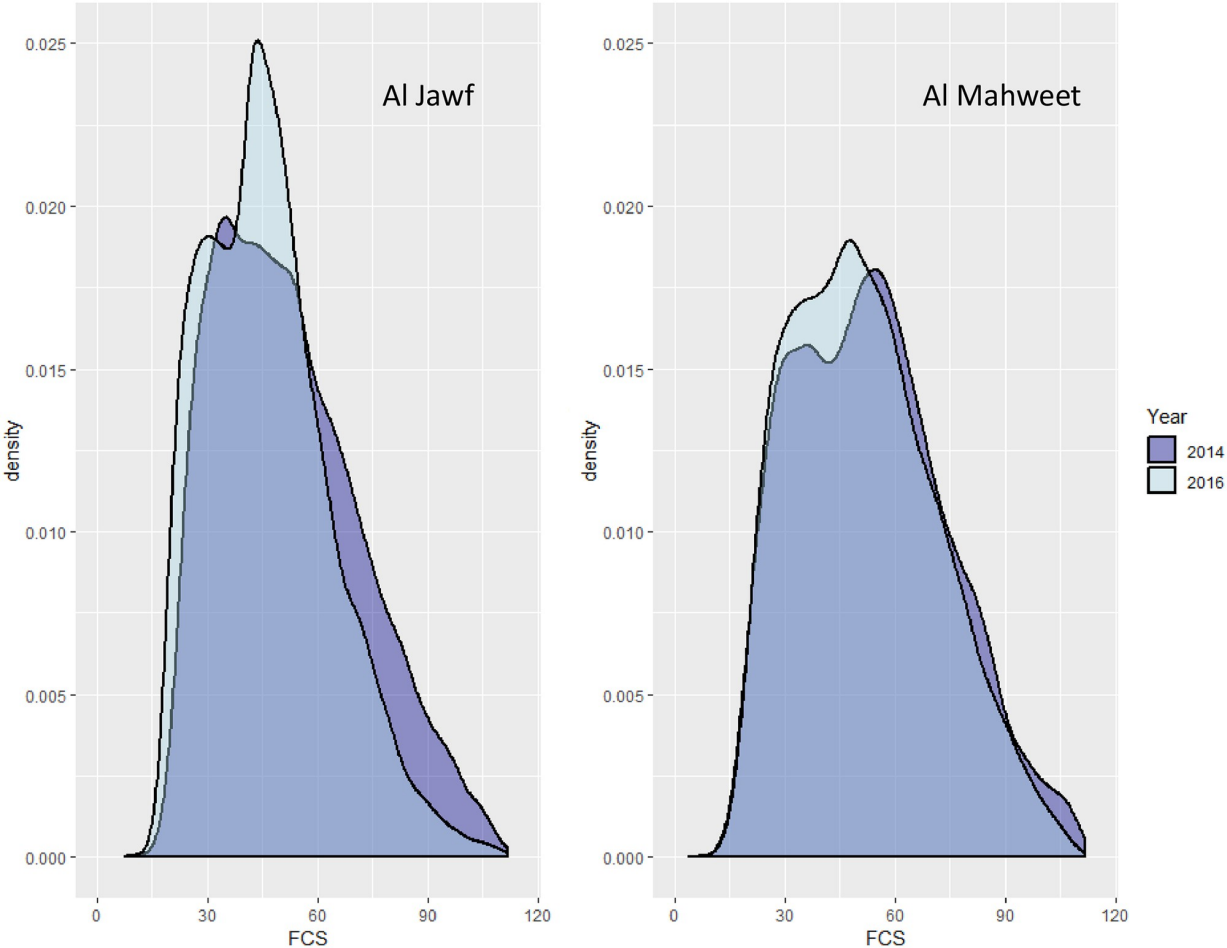
Density curves for simulated FCS distributions from fitted models in Al Jawf and Al Mahweet.

We next consider the region of Al Jawf, where food security levels in 2014 were considerably better than the national average, with 10.4% of surveyed households reporting consumption below minimal levels (FCS ≤ 28) and 35.8% below acceptable levels (FCS ≤ 42). The median household consumed foods from 9 of the 12 HDDS categories per week, but after frequency weighting this was reduced to 6. Bread, oil, and sugar were consumed daily by over 98% of the households, while fish was only consumed by 2 of the 480 households in this inland region. Strong pairwise correlations (${\hat{\rho }}_{jk}>.5$) were estimated for consumption of fruit, pulses, meat, and eggs, while potatoes, rice and vegetables were also correlated. Consumption of some food groups was highly dispersed. For example, while the median value for dairy was 2 days, 40% of households consumed no dairy at all, 30% consumed dairy on 1-3 days, and the remaining 30% consumed dairy 4-7 days. Similarly, the daily probability of consumption for eggs was estimated to be 0.055, which, for a standard binomial model would imply that consumption on 4 or more days would be extremely unlikely to observe in any households. However, in Al Jawf 11% of households reported this level of consumption. In 2016, the percentage of households with FCS at or below 28 increased to 16.4% (*p*-value = 0.01) while the proportion below the acceptable level also significantly increased to 46.7% (*p*-value = 0.002). Overall dietary diversity declined to 8 food groups per week, and the median frequency weighted HDDS declined to 5.3 per day. However, unlike in Ibb, food assistance was widely available in Al Jawf in 2016, where 50% of the 360 households surveyed were recipients of food assistance with the primary items provided being wheat (48 households), beans/pulses (154 households), and oil (88 households). In 2014, no such assistance had been provided in this region. As a likely consequence of food assistance, daily bread and oil consumption were not diminished in this region and the proportion of households consuming pulses on a daily basis increased from 6% to 36%. However, variability also significantly increased, and 30% of households did not consume any pulses during the 2016 survey period. Among the other food groups there were significant declines in consumption of tubers, vegetables, fruits, dairy, and meat, and variability significantly increased for rice and dairy products. Changes in the estimated correlations between food groups indicate a distinction between products only available through the standard market system and those also provided through assistance (bread, pulses, oil) or self-production (dairy), and the results suggest that recipients of pulses via food assistance did not increase their consumption of any other food groups (see Fig 5, upper panels). Thus, the multivariate modeling reveals an overall contraction of the diet for residents in Al Jawf with increased variability across households (see Fig 6, left panel), while the impact of food assistance on consumption appears to have been to enable a minimal diet for recipients largely consisting of the provided items.

In comparison we consider Al Mahweet, the only region of the 19 where there was no significant decline in food security between 2014 and 2016. Food security was comparable to Al Jawf in 2014, with 11.7% below minimal consumption levels in 2014 and 34% below acceptable levels. The unweighted median HDDS was 9 and the weighted median score was 6. All food groups other than fish and eggs (which were only sporadically consumed) had estimated daily consumption probabilities of 0.10 or higher, and bread, oil, sugar, and condiments were all consumed daily. Potatoes, rice, fruit and meat were all highly correlated (${\hat{\rho }}_{jk}>0.50$) and dairy consumption was widespread with a large proportion of households (23%) reporting their own dairy production. Unlike in Al Jawf, in 2016 only about 9% of Al Mahweet households received food assistance, primarily in the form of beans/pulses (6.7%) and/or oil (3.6%). Estimated consumption probabilities for potatoes, rice, and condiments dropped sharply (all by over 30%), and there was also a 5% drop for oil. However, the probability of egg consumption increased by 0.05, that for beans/pulses increased by 0.08, and there were no significant shifts in any other food group. For this reason, there was little change in the FCS score due to the relative weights of these groups: the proportion of severely insecure households in 2016 was essentially constant 13.3% (*p*-value = 0.53) and the proportion of households with inadequate consumption increased slightly to 40.0% (*p*-value = 0.08). Density plots for the modeled FCS are shown in the right panel of Fig 6. Nonetheless, dietary diversity did decline: the median unweighted HDDS score was reduced from 9 to 8, and the weighted score dropped from 6 to 4.9. A major question of interest, then, is how Al Mahweet was largely able to maintain its 2014 levels of food security, even in the absence of assistance, while Al Jawf was unable to do so even with widespread food aid.

The key difference between the two regions appears to be the self-production of agricultural products in Al Mahweet that enabled residents to mitigate the effect of market price increases. Based on survey data, the percentage of households producing their own bread, eggs, and dairy were 12.5%, 8.6%, and 22.9%, respectively, compared with only 1.7%, <1%, and 8.3% in Al Jawf. This likely explains why fruit, typically considered a “luxury” item in times of crisis, was highly correlated with both eggs and dairy in Al Jawf but was uncorrelated with either in Al Mahweet (see Fig 5). Furthermore, dairy consumption explained over half of the variation in the FCS scores in both 2014 and 2016, with 92% of the households in Al Mahweet that met the FCS threshold for acceptable consumption reporting some consumption of dairy products (compared with 41% in Al Jawf).

### Shabwa and Ad Daleh

In the regions of Shabwa and Ad Daleh food security was already very poor in 2014, with 31.5% and 29.2% scoring below 29 for the FCS, and 57.3% and 54.8% scoring below 43, respectively. The empirical FCS distributions for the two regions were statistically indistinguishable (*p*-value = 0.36, K-S test), and both were highly right-skewed. The median HDDS for any weekly consumption was 8, but only 5 after frequency weighting in both regions. In Shabwa, we again observed daily consumption of bread, oil, sugar, and condiments, and rice was also consumed at very high levels (${\hat{p}}_{j}=0.95$), but estimated probabilities for daily consumption for all other food groups were below 0.3 with estimates for fruit, eggs, and fish all below 0.05. High pairwise correlation parameters for random effects (${\hat{\rho }}_{jk}>.5$) were estimated for tubers, vegetables, fruits, beans, eggs, dairy, and meat, presumably reflecting the habits of the few households with resources to afford a diverse diet. Similar patterns were seen in Ad Daleh, although seafood consumption was negligible in this inland region and the consumption of potatoes, rice, and vegetables was estimated to be lower and/or more variable.

In 2016 similar numbers of households reported receiving food assistance, 65/360 (18%) in Ad Daleh and 47/360 (13%) in Shabwa. However, the nature of the assistance was considerably more limited than for Al Jawf, with fewer than 5% of households receiving wheat or rice. 29 and 31 of the 360 surveyed households received pulses in Ad Daleh and Shabwa, respectively, and 42 and 9 households received oil. Food security plummeted in both governorates. The proportion of severely food insecure households increased to 43% in Shabwa and 56% in Ad Daleh, with 86% and 85% of households below the FCS threshold for acceptable consumption in the two regions, respectively. Given that in-kind assistance was essentially the same in the two regions, why was the increase in severe food security so much greater in Ad Daleh? A comparison of the fitted models indicates few differences, with Shabwa actually having lower estimated probabilities of consumption for pulses and sugar and a significantly lower median weighted HDDS score based on model simulations (see Table 2). However, there was a sizeable increase in egg consumption in Shabwa, largely due to a marked investment in self-production (reported by 14% of households, compared with fewer than 1% in 2014). In the FCS calculation, eggs provide a “high value” source of protein and largely explain the difference in severe insecurity between the two regions, as 84% of households above the threshold for severe food insecurity in Shabwa reported consumption of eggs at least once in the previous 7 days, compared with only 32% of these households classified as severely food insecure. This is in sharp contrast to 2014, where any egg consumption was only reported by 24% of households (88% of whom were classified as having acceptable consumption) and these were all purchased with cash or credit. In 2016, eggs were no longer positively correlated with rarely consumed food groups such as meat or beans, and their consumption was more evenly reported by both low- and high-consumption households as indicated by the significant reduction in the estimate for *σ*_*j*_.

**Table 2:** Parameter estimates for Shabwa and Ad Daleh in 2014 and 2016.

Food Group	Year	Proportion *p*		Dispersion *θ*		Standard Deviation *σ*	
		Ad Daleh	Shabwa	Ad Daleh	Shabwa	Ad Daleh	Shabwa
Bread	2014	0.99	1.00	0.81	3.18	0.56	2.14
	2016	0.99	0.95 ↓ *	1.08	6.81 *	1.90	1.21
Potatoes	2014	0.10	0.20 *	12.21	19.00	1.83	1.08 *
	2016	0.02 ↓	0.03 ↓	12.12	19.09 *	1.71	1.99 ↑
Rice	2014	0.64	0.95 *	5.08	2.91	2.57	2.38
	2016	0.26 ↓	0.83 ↓*	4.31	4.60	2.20	1.54
Vegetables	2014	0.21	0.28 *	11.18	19.55	1.41	1.02 *
	2016	0.05 ↓	0.05 ↓	11.73	19.99 *	1.33	1.64 ↑
Fruit	2014	0.07	0.04	16.40	12.41	1.10	1.57
	2016	0.01 ↓	0.00 ↓	16.82	12.33	1.60	2.23
Pulses	2014	0.19	0.11	4.96	6.24	2.08	2.11
	2016	0.10 ↓	0.05 ↓*	4.22	5.82	1.62	1.46
Eggs	2014	0.02	0.02	6.98	10.96	1.81	1.84
	2016	0.02	0.20 ↑*	6.33	10.27	1.32	0.87 ↓
Dairy	2014	0.04	0.09	3.18	4.25	3.66	1.85
	2016	0.03	0.01 ↓	2.74	4.38	1.61	1.40
Meat/Poultry	2014	0.14	0.15	17.95	18.43	1.04	0.97
	2016	0.05 ↓	0.04 ↓	17.43	19.44	1.59	1.22
Fish/Seafood	2014	0.00	0.05 *	7.64	8.46	0.81	1.05
	2016	0.01	0.02	7.97	8.57	0.96	1.49
Oils/Fats	2014	0.99	1.00	3.27	1.83	3.87	3.53
	2016	0.96	0.90 ↓	3.66	5.66 ↑	2.99	1.54 ↓*
Sugar	2014	0.99	1.00	0.75	3.57	1.59	1.85
	2016	0.96	0.90 ↓*	2.44 ↑	6.79 *	1.92	1.46
Condiments	2014	0.98	0.99	3.48	3.84	3.45	2.70
	2016	0.97	0.93	3.42	4.07	4.03	3.86

Asterisks (*) indicate non-overlapping 95% HPD intervals for the two regions, while non-overlapping 95% HPD intervals across years within each region are indicated by arrows for increasing (↑) or decreasing (↓) estimates.

### Hadramout

**Fig 7:**
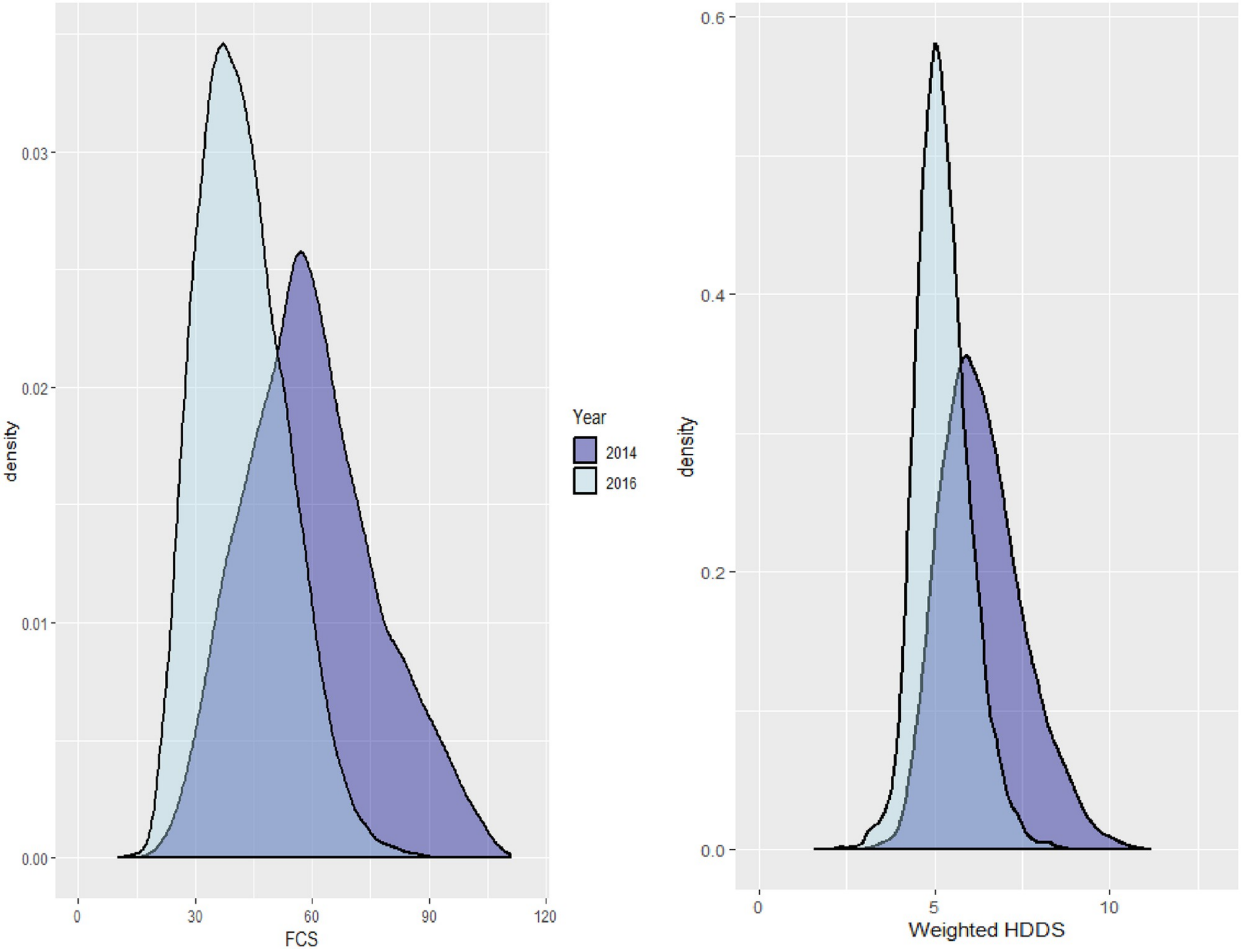
Simulated FCS and weighted HDDS densities from fitted models for Hadramout in 2014 and 2016.

In this relatively prosperous region, only 4.6% of households were severely food insecure in 2014 with 12.9% below the threshold for acceptable consumption. Dietary patterns were varied, with daily consumption probability estimates greater than 0.10 for all food groups. Bread, sugar, oils, and condiments were consumed daily, and seafood, vegetables and beans were all consumed several days per week by the majority of households. Animal proteins such as meat/poultry, dairy, and eggs were less commonly consumed, but given the high levels of seafood consumption in this coastal region this appeared to be largely a matter of dietary preference. The median unweighted HDDS in Hadramout was 10 and after weighting this was only reduced to 6.3, notably higher than any other region considered in this analysis. Furthermore, the distributions for the FCS and weighted HDDS scores were unimodal and largely symmetrical, indicating less economic variability in this region than observed elsewhere (see Fig 7). Two years later, the situation had changed dramatically. In 2016 the proportion of severely food insecure households more than doubled to 11.1%, and the number of households below the threshold for adequate consumption rose to 62.5%, a staggering increase. The median unweighted HDDS score declined to 8, and the median frequency-weighted HDDS was reduced to 5.07, similar to several of the poorer regions previously discussed.

**Fig 8:**
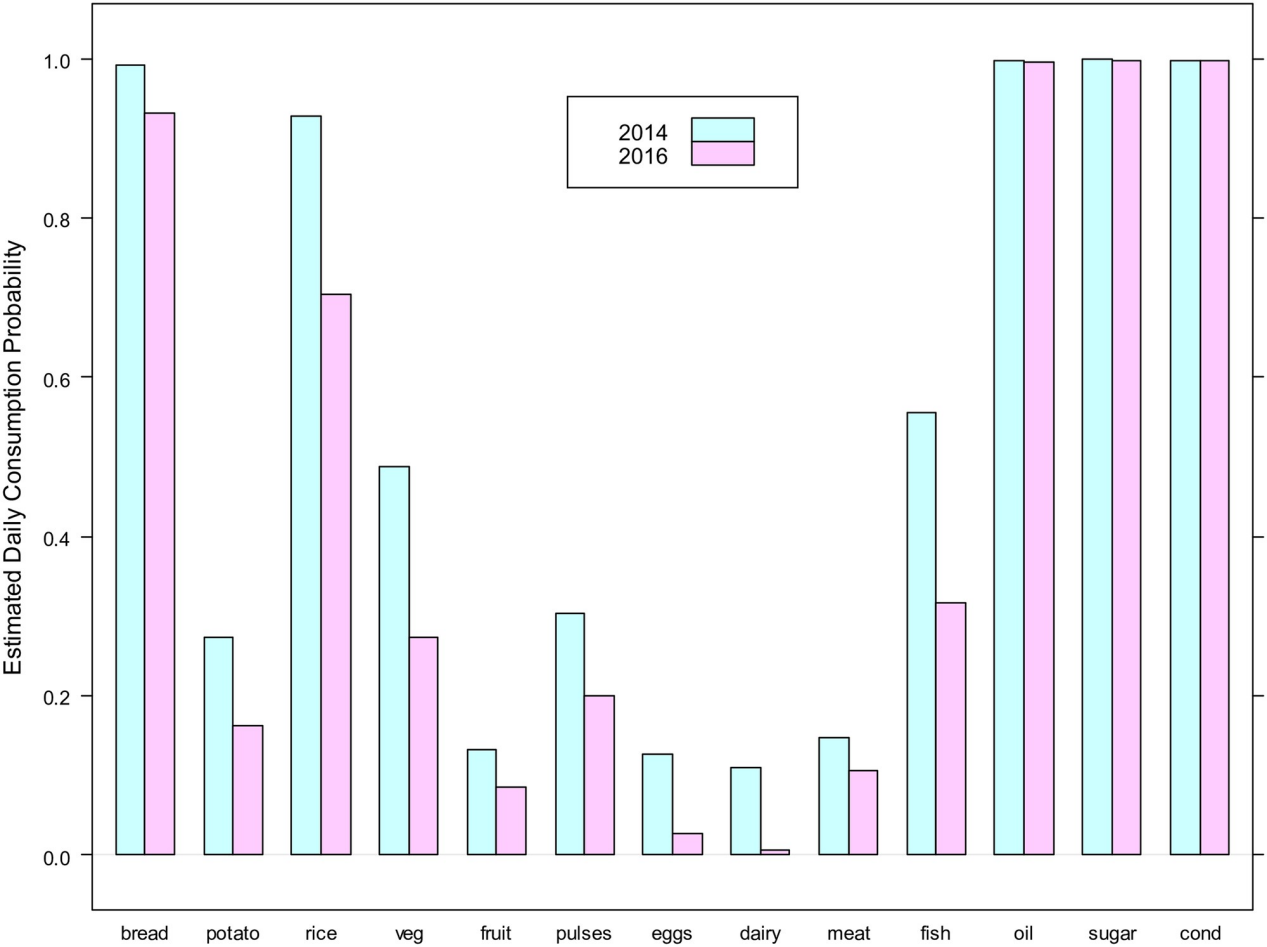
Estimated food group beta-binomial parameters *p_j_* for Hadramout.

Parameter estimates significantly decreased from 2014 for every food group other than oil, sugar, and condiments, with the largest declines observed for fish (0.23), rice (0.21), and vegetables (0.20). Even wheat/bread consumption was reduced by 0.06, a phenomenon not seen in other regions. Interestingly, there were no significant shifts in dietary patterns in that the relative ordering of consumption frequency estimates was unchanged, indicating that most households chose to deal with the crisis by reducing their consumption overall rather than shifting towards a more limited daily diet (see Fig 8). Estimates for dispersion and random effects terms also indicated few differences, although there were notable increases in the correlation estimates for the pairs {pulses, rice} and {meat, fish} from nearly 0 in 2014 to over 0.5 in 2016. Because both food assistance and self-production of any kind were negligible in Hadramout (reported by fewer than 7% and 5% of households respectively), the changes in consumption levels can be almost completely explained by impacts of the conflict on markets and the local economy.

## Discussion

Fitted Bayesian models, by definition, are generally heavily dependent on the choice of prior distributions for model parameters, and the importance of choosing such priors carefully is often overlooked in practice. The food consumption setting is one in which Bayesian assumptions are generally well met, and the use of priors derived from baseline models is not only intuitively reasonable but also has the desired effect of reducing variability in models estimated from emergency surveys. However, we have found that the use of flexible weights to adjust the variance of the prior distributions for the parameters *p*_*j*_ and *θ*_*j*_ is a critical component of our approach. Given the large sample sizes typically encountered for CFSVAs and other national or regional household assessments such as the Living Standards Measurement Survey (LSMS), the baseline posterior distributions for these parameters are generally quite narrow, and without increasing the variance of these distributions to fit the updated models the bias towards the prior is generally too great to yield any statistical evidence of dietary change even when the data strongly suggest it. This is of particular concern for the dispersion parameters *θ*_*j*_, as the highly skewed Gamma distribution is strongly biased toward the prior mean unless the prior variance is increased using very low weights. Similarly, truncated normal priors for correlation terms should also be moderately diffuse. While our asymptotic results can provide some guidance as to the expected bias in the modes of posterior distributions given fixed parameter values and choices of prior weights, we have also found that goodness of fit tests are needed to ensure that simulated observations from our fitted models are reasonably consistent with, although generally smoother and less variable than, the emergency survey data from which they are estimated. Refining our approach for the fitting and evaluation of updated models for implementation by the food security community is an ongoing research direction. Another extension of our model under development is the addition of covariates at the household or community level. These could include factors such as gender of the head of household, primary livelihood, and proximity to fresh water, among many others regularly considered by food assistance organizations.

An important objective in fitting updated consumption models is to provide quantitative evidence for dietary change. With respect to statistical inference, with appropriate prior weighting our updated Bayesian models are highly sensitive to changes in the parameters *p*_*j*_, *σ*_*j*_, and *ρ*_*jk*_ from the baseline to the emergency rounds, although significant shifts in *θ*_*j*_ are quite difficult to detect due to the wide HPD intervals associated with these dispersion parameters. Some uncertainty in these parameters is generally not a significant concern from a modeling standpoint, but for food consumption analysis it is important to distinguish between a standard binomial distribution with parameter *p*_*j*_, for which the majority of households are associated with consumption close to the mean, and the U-shaped or highly skewed distribution associated with the same value of *p*_*j*_ that reflects consumption inequality or inherent variation in dietary habits. Dispersion in consumption has generally been disregarded in food security analyses despite a high prevalence of survey results in which the most frequently reported values are “0” and “7” for individual food groups. An important contribution of our beta-binomial modeling framework, therefore, is to provide quantitative estimates of this type of variability so that food security organizations can better understand consumption patterns in their beneficiary communities and provide more meaningful reports of assistance impacts to donors and partners.

As our results and underlying data demonstrate, the quality and diversity of the diet in Yemen steeply declined for the majority of residents between 2014 and 2016, and conditions have further deteriorated since that time. Because nearly all of the country’s food is imported, most Yemenis are highly dependent on public markets, and the effects of logistical constraints on food distribution combined with the country’s financial collapse have led to dire consequences for the majority of households [26], most recently exacerbated by the COVID-19 pandemic, natural disasters, and ongoing conflict [27]. Interestingly, our results indicate that despite these hardships most Yemenis sought to maintain their standard diet during the initial crisis period, simply buying “less of everything” and reducing their overall consumption frequencies rather than making any major dietary shifts. Where such shifts were present, they were generally due to the effects of food assistance (pulses in particular) or an increase in self-production of items such as dairy and eggs. Another clear consequence of the crisis was a great reduction in consumption inequality, particularly in large urban areas such as Ibb, as there were far fewer households with the buying power to sustain a highly diverse diet as conditions continued to deteriorate. In our models, this was generally reflected through the estimated random effects covariance matrix, with reductions in both the variance parameters *σ*_*j*_ and the magnitude of pairwise correlations *ρ*_*jk*_.

Summary statistics including the FCS and HDDS clearly reflected these trends, however as aggregate indicators they are unable to explain the ways in which dietary behavior is contributing to changes in household scores. A few studies have explored the variability in consumption patterns and limitations in how these proxy food frequency-based measures reflect dietary adequacy and changes in diet. For example, analytical work in Mozambique comparing food frequency-based measures to a detailed 24-hour recall of food consumption showed regional differences in food frequency thresholds for classifying dietary adequacy [28]. Furthermore, an important consideration with both the FCS and the HDDS is their inherent bias towards the consumption of animal proteins. While an individual consuming a daily “fast food” diet of bread, potatoes, meat, dairy, fats and sugar would score a 91 on the FCS and and 8 on the HDDS, a vegan consuming foods from only plant-based sources would score a maximum of 56 on the FCS and still an 8 on the HDDS, despite eating a much more varied diet containing fruits and vegetables. This is not only an important issue for sustainable development [29], but also for the monitoring and evaluation of food assistance programs.

Particularly important for humanitarian assistance efforts is the role of consumption-based indicators in monitoring the impact of programs. Food items provided via direct assistance in Yemen (and in most other WFP programs) are typically limited to grains, pulses, cooking oil, and sugar. A household consuming only these items on a daily basis would not exceed the threshold for acceptable consumption (earning an FCS score of 42 and a HDDS score of 4), which may be considered an operational failure despite the fact that such assistance can have a sizable impact on the ability of the household to avoid severe malnutrition and reallocate scarce resources to other expenses. In its 2016 project report on the Emergency Food Operation in Yemen [30], WFP stated a target goal of reducing the percentage of severely food insecure households to below 12.4%, and reported a remarkable decrease from 62% at the start of the operation in June 2015 to 18.5% by December 2016 in beneficiary communities. However, during this time the percentage of households with borderline FCS scores increased from 29% to 34%, far exceeding the target goal of under 5.8%. This is to be expected, as many of those households that received assistance improved from severely to moderately food insecure (particularly given the partial rations provided in some communities), while households that were already moderately food insecure would be unlikely to qualify for assistance given the extent of need. Rather than relying only upon indicator thresholds, a more comprehensive analysis of dietary shifts using our multivariate approach provides a better evaluation of the effectiveness of food assistance in emergency situations. This is clearly illustrated by the apparently contradictory results in Al Jawf and Al Mahweet. While the former received large amounts of assistance, FCS thresholds indicated a greater decline in food security than in the latter, where almost no assistance was received. Upon further analysis our results demonstrated that, while dietary diversity was, in fact, lower in Al Mahweet in 2016, many households maintained higher FCS scores solely through dairy consumption enabled by self-production. The multivariate beta-binomial models presented here provide a means to explain and contextualize changes in aggregate indicators based on consumption modules, allowing for more nuanced and insightful decision-making by aid agencies who wish to optimally allocate scarce resources and progress towards meeting the ultimate goal of Zero Hunger worldwide.
